# Care inequality: care received according to gender, marital status, and socioeconomic status among Korean older adults with disability

**DOI:** 10.1186/s12939-019-1008-0

**Published:** 2019-07-03

**Authors:** Soong-nang Jang, Ichiro Kawachi

**Affiliations:** 1 Red Cross College of Nursing, Chung-Ang University, 84 Heukseok-ro, Dongjak-gu, Seoul, 06709 South Korea; 2 Department of Social and Behavioral Sciences, Harvard T.H. Chan School of Public Health, Boston, Massachusetts USA

**Keywords:** Caregiving, Inequality, Gender, Marital status, Socioeconomic status

## Abstract

**Background:**

We sought to identify the types of care and care resources available to older Korean adults with disabilities, and document the inequality in care received according to gender, marital status, and socioeconomic status.

**Method:**

Data were derived from the sixth wave of the Korean Longitudinal Study of Ageing. The sample consisted of 946 men and women who were disabled in ADL and IADL. Generalized linear models and analyses of covariance were used to evaluate group differences in types of care received and care resources. The outcome variables were main primary caregivers, the total number of available caregivers, hours of care received per day, number of days of care, and fees paid to caregivers.

**Results:**

In total, 41.7% of men with ADL/IADL disabilities reported that they did not receive formal or informal care from any source, compared with 30.7% of women. Almost half (49.2%) of men without a spouse were in a state of care deficit (vs. 30.8% in women without a spouse, *P* < 0.001). Among care recipients, men reported receiving higher average days of care per month than women (25.6 vs. 21.2 days, *P* < 0.01). Both men and women received care primarily from their spouse, but adult children were more frequently care providers for older women than men. A combination of care from spouse and paid caregiver was more frequent among women. Dependent older people with high household incomes had a higher likelihood of receiving care There was the clear gradient in rate of paid formal caregivers use by household income (higher income = higher use) among women but not men.

**Conclusions:**

Care types and resources among disabled older adults appeared to be different by gender, marital status and socioeconomic status under the cultural phenomenon and contextual circumstances in the aging Korean population.

## Introduction

Care is not simply about personal preferences, but rather is also about inequalities that penetrate deeply into society. Demographic and socioeconomic factors shape a person’s life experiences long before he or she requires long-term care. Group differences in care behaviors manifest in a variety of ways. Thus, it is necessary to discuss care not only in socioeconomic terms, but also in the context of national, ethnic, and cultural differences [1–3]. Although many studies corroborate the caregiving profiles, inequalities in the care received by those who need it in specific social and cultural settings is a more complicated issue that requires further analysis empirically.

It is important to consider the impact of both personal and environmental characteristics on a variety of outcomes among persons receiving care [4]. A review of the literature suggests that personal characteristics (e.g., gender, race, marital status, functional status, dementia, and the availability of family members) and environmental characteristics (e.g., availability of formal caregivers, infrastructural investments in nursing homes, insurance systems for long-term care) are important predictors of the receipt of formal and informal care [4–8]. For example, in the absence of available family members, people are more likely to receive formal assistance [8]. Focusing on psychosocial factors, such as satisfaction of care, is insufficient to understand the care choices of a given population [9].

In Korea, as in other East Asian countries, long-term care policy is still dominated by the Confucian model (i.e., reliance on family caregiving as the main bulwark of long-term care). This is reflected in the low level of investment in long-term care infrastructure (e.g., an informal family caregiver support system including carer allowance, policies governing compatibility of caregiving with work, and a care-giving workforce) the inadequate social security safety net (to enable seniors to live independently of their adult children) [10–12], and the cultural stigma associated with having one’s parents or spouse cared for by strangers.

Group differences in care behaviors manifest in a variety of ways. Provision of care is typically considered to be a “feminine”’ domain, and much research has examined gender incongruence and role theory in this context [13–15]. Gender differences in the provision of care, and segregated gender roles in caregiving, are common phenomena in both Western and Eastern countries. However, a more important and difficult problem—how to secure the best care and from whom—faces contemporary older Koreans who experienced gender segregation under the strong patriarchal culture since childhood [16, 17]. An international comparative study of informal caregivers suggested that the proportion of female caregivers was higher in Spain (66.7%) and Korea (64.8%) compared with Denmark (36.9%) and Sweden (35.0%) [18].

The impact of marital status, with respect to access to formal and informal care, has been indicated by community-based studies showing greater rates of family support among married persons [19], or a lower rate of formal care service use among the married [20], as well as gender differences in marital status effect on care use [14]. Previous research also suggests that those without available family members are more likely to receive assistance from formal sources [5].

Children providing care for their older family members have increasingly met with challenges, owing to the strain imposed by widespread job instability, multiple national economic crises, and an increase in women’s social participation. The dearth of available care, synergistically resulting from the dominant Confucian culture and the economic instability of the Korean family unit, may be responsible for the unequal distribution of care resources. The role of the caregiver is increasingly being fulfilled by family members, such as older women, who are already excluded from the labor market [21].

Until recently, most previous research on caregiving has been conducted from the perspective of the unpaid informal caregivers. If we only focus on analyzing unpaid family caregivers, that assumes that all caregivers are intimate care providers [9, 22]. This induces to concerns on methodological individualism [9]. Not all care should be analyzed as results of individual behaviors. To detect inequalities in care more precisely, it is necessary to include a wide range of variables relevant to care, including the gender, marital status, and socioeconomic status of the cared-for person. Any such investigation must go beyond existing research on the nature of care, which to date has focused only on the identity of the major informal caregivers [4–8] or the prevalence and associated factors of unmet care needs [23]. Research that covers both the imbalance in care resources and inequalities in the care received is needed, including the variables of type of care (formal or informal), care received from a number of sources, the number of hours of care per day and number of days of care per month, and fees paid to caregivers.

This study was conducted to identify the care types and care resources available to older people with disabilities in daily activities, and to investigate whether they vary by gender, marital status, and socioeconomic status. We discuss the inequalities in care present in Korean society, in the context of both a rapidly aging population and a rapidly increasing number of people who need care but are still dependent on their families to provide it, even though family resources are increasingly scarce. This study will help stimulate research on imbalances in care resources, bring more focus to these issues and how they affect different populations.

## Methods

### Study design and data

We used cross-sectional data from the sixth wave of the Korean Longitudinal Study of Ageing (KLoSA), conducted by the Korea Employment Information Service in 2016. The KLoSA is a national representative longitudinal study that follows up its respondents in every even year (from 2006 onwards). The KLoSA targeted Koreans aged 45 years or older and their families. Households were selected using multistage stratified probability sampling based on geographical area. Trained interviewers conducted face-to-face interviews using CAPI with each panel participant when at least one eligible family member in the household was aged 45 years or older. More detailed information is available at the KLoSA website (http://survey.keis.or.kr/klosa/klosa01.jsp). The sample recruited to this study was limited to adults with one or more disabilities in activities of daily living (ADLs) or Instrumental Activities of Daily Living (IADL). Age-adjusted disability rate of KLoSA participants aged 65 or over in 2016 (wave 6) were 12.9% in men and 7.9% in women. Men had higher rate of disability rate because men showed high dependency in the specific IADL items, such as doing laundry, doing house chores and preparing meals. The rate of disability in these three items was about 6 times higher than that of women. A total of 946 persons (14.3% of the total sample of 6618), consisting of 463 men and 483 women, exhibited one or more ADL or IADL scale disability. All 946 selected participants were included in the final analysis.

### Measures

#### Dependent variables

Nine dependent variables were included in the analysis, as follows: (1) the main primary caregivers among all types of formal and informal caregivers, (2) any additional caregivers, (3) number of caregivers (including formal and informal), (4) days of care received per month, (5) hours of care received per day, (6) use of a paid formal caregiver, (7) total amount paid to caregivers per month, (8) total fees paid by the cared-for person to caregivers, and (9) total fees paid by family members to caregivers. These data were obtained using questions regarding the source of assistance of persons who reported receiving help with ADL/IADL tasks. The response options for the source of care included spouse, children, son-in-law, daughter-in-law, grandchildren, siblings, parents, other relatives, friends, neighbors, and private or publicly employed formal caregivers. Respondents indicated the top five caregivers who provided the most assistance.

The first dependent variable, main caregivers, constituted a categorical measure of the caregivers who provided the most primary assistance: spouse, children, other relatives or friends, or paid formal caregivers. If no assistance was received from any formal or informal source, the response was coded as none (care deficit). Care deficit has differently been measured in aging research. In this study, care deficit was defined 1) does not perform independently more than one item of ADL and IADL, and 2) receives no human assistance but reports needing help. The second dependent variable, additional caregivers, was categorized as one of the following 11 combinations of main caregiver plus additional caregiver: spouse only, spouse + unpaid informal caregiver, spouse + paid formal caregiver, child caregiver only, child + unpaid informal caregiver, child + paid formal caregiver, other relatives or friends only, other relatives or friends + unpaid informal caregiver, other relatives or friends + paid formal caregiver, paid formal only, and paid formal + unpaid informal caregivers. The third dependent variable, number of caregivers, indexed the number of people who provided formal or informal care to older adults with disabilities, excluding those who did not receive help. The fourth dependent variable, caregiving days per month, measured the total number of days on which help was received from each caregiver per month, and the fifth variable, caregiving hours per day, was the total number of hours of care received from each caregiver per day. The sixth dependent variable, payment to caregivers, was dichotomized: a “yes” response indicated that the dependent older adult received care from a formal paid caregiver. The last three dependent variables concerned costs of care, i.e., the total amount paid to the caregiver during the past month, the amount paid by the cared-for person directly to the caregiver during the past month, and the total amount paid by the family members to the caregiver during the past month.

#### Independent variables

The personal characteristics assessed included demographic factors, such as age, gender, marital status, residential region, living arrangement, educational level, household income and personal income. Age (years) was represented by dummy variables for 54–64, 64–74, 75–84, and 85 and over. Marital status was represented by dummy variables for married and living together (1), separated, divorced, widowed, and others (0). Region was dichotomized as living in rural area (0) or in urban area (1). Living arrangement was categorized as living alone, living with spouse only, or living with others. Educational level was categorized as elementary school or less, middle school graduate, or high school graduate or higher. Household equivalized income was calculated as total household income divided by the square root of the number of household members; these scores were then divided into tertiles. Personal income per year was also divided into tertiles.

For comparative analysis of care source by gender, marital status and socioeconomic status, we included covariates that could influence caregiving patterns, such as self-rated health, functional status, cognitive function, chronic diseases, number of chronic diseases, health insurance, and long-term care service use. To determine self-rated health, respondents were asked to rate their overall health as either excellent, good, fair, poor, very poor, dichotomized as good (excellent, good or fair) or poor (poor, or very poor). Physical functional status was measured using the 7-item ADL scale and the 10-item IADL scale. The ADL scale includes items on getting dressed, washing the face and hands, bathing, eating meals, leaving a room, urination, and defecation, and the IADL scale includes personal grooming, going out for short walks, using transportation, making/receiving phone calls, managing money, doing household chores, preparing meals and cooking, shopping, taking medications, and doing the laundry. If respondents were dependent for a given activity, they were categorized as having a deficit (deficit = 1, other = 0) therein: sum of ADL and IADL deficit scores are representative of physical functional status (ranged from 1 to 17). For the cognitive functional status, the Korean version of the Mini-Mental State Examination (K-MMSE) was applied and the score determined according to the guidelines for the standard MMSE. The K-MMSE score was categorized as normal (24–30), mild cognitive disorder (18–23), or moderate-to-severe cognitive disorder (0–17). The chronic disease prevalent status and the number of chronic diseases reflects the history of diagnosis (by a physician) of the following eight conditions: hypertension, diabetes, cancer, lung disease, heart problems, stroke, arthritis, and gastrointestinal disease.

Generally, all respondents were eligible for national public health insurance, regardless of morbidity and disability. Those in poverty belonging to a household without any employed family members were covered by medical aid; thus, the type of insurance was categorized as health insurance or medical aid. The use of long-term care services (provided by national long-term care insurance) was explored by a dichotomous variable pertaining to whether or not the dependent older adult was enrolled in a program involving home care, daycare, assistive devices, or other forms of care paid for in part by national long-term care insurance.

### Statistical analysis

We calculated frequencies, proportions, and means (± SD) of demographic, socioeconomic, health, and functional status variables according to gender for our entire dependent older adult population (*N* = 946). We used chi-square tests and t-tests to compare the distributions of these variables by gender (Table 1). The first part of the data analysis addressed the first research objective, i.e., to examine gender differences in care received according to nine dependent variables. We used a generalized linear model to explore binary categorical outcomes, including the primary caregivers, additional caregivers, using paid formal caregivers after adjusting for the age, marital status, region, socioeconomic status, health and functional status, type of medical insurance, and long-term care enrollment status of the cared-for person. Family size and each of chronic diseases were excluded in the final analysis because there might be redundant to living arrangement and the number of chronic diseases, furthermore, there were no association with care-receiving outcomes. Using the subgroup those who received care (*n* = 626), analyses of covariance (ANCOVAs) were also performed to test gender differences in the numeric outcomes such as the number of caregivers, number of care hours. The cost of providing care was calculated only for those who received paid care (*n* = 66, excluding 32 cased answered not known among 98 paid caregiver users). Least square means and adjusted estimated rate data are presented in Table 2.

**Table 1:** General characteristics and health status among dependent older adults in KLoSA (*N* = 946)

	Total N (%) or Mean ± SD	Gender	
		Men	Women
Total	946 (100.0)	463 (48.9)	483 (51.1)
Age (years), Mean ± SD	77.99 ± 10.44	73.84 ± 10.40	81.96 ± 8.82**
< 65 years	132 (14.0)	106 (22.9)	26 (5.4)
65–74 years	172 (18.2)	116 (25.1)	56 (11.6)**
75–84 years	362 (38.3)	167 (36.1)	195 (40.4)
85+ years	280 (29.6)	74 (16.0)	206 (42.7)
Marital status			
Widowed, separated, divorced or others	360 (38.1)	46 (9.9)	314 (65.0)**
Married	586 (61.9)	417 (90.1)	169 (35.0)
Living arrangement			
Living alone	151 (16.1)	14 (3.1)	137 (28.5)**
Living with partners only	371 (39.5)	254 (55.3)	117 (24.4)
Others	417 (44.4)	191 (41.6)	226 (47.1)
Region			
Urban	664 (70.2)	345 (74.5)	319 (66.0)**
Rural	282 (29.8)	118 (25.5)	164 (34.0)
Educational level			
Elementary school or below	571 (60.4)	161 (34.8)	410 (84.9)**
Middle school graduated	119 (12.6)	80 (17.3)	39 (8.1)
High school or over	256 (27.1)	222 (47.9)	34 (7.0)
Personal income level^a^	930.26 ± 1897.87	1312.81 ± 2173.53	561.45 ± 1499.98**
Low (< 270)	310 (33.4)	113 (24.8)	197 (41.6)**
Middle (270–640)	310 (33.4)	134 (29.4)	176 (37.2)
High (640+)	309 (33.3)	209 (45.8)	100 (21.1)
Household income level^a^	1357.13 ± 1250.01	1488.48 ± 1408.79	1231.03 ± 1062.07**
Low(< 636)	315 (33.8)	131 (28.7)	184 (38.7)**
Middle(636–1443)	300 (32.2)	160 (35.0)	140 (29.4)
High (1443+)	318 (34.1)	166 (36.3)	152 (31.9)
Self-rated health, Mean ± SD	3.87 ± 0.90	3.65 ± 0.950	4.07 ± 0.816**
Poor	877 (92.7)	412 (89.0)	465 (96.3)**
Good	69 (7.3)	51 (11.0)	18 (3.7)
MMSE, Mean ± SD	19.67 ± 8.04	22.68 ± 7.24	16.54 ± 7.64**
Dementia	297 (35.9)	84 (19.9)	213 (52.5)**
Cognitive declining	193 (23.3)	86 (20.4)	107 (26.4)
Normal	338 (40.8)	252 (59.7)	86 (21.2)
National insurance type			
Health insurance	846 (89.4)	417 (90.1)	429 (88.8)
Medical Aids	100 (10.6)	46 (9.9)	54 (11.2)
Long term care service use			
No	890 (94.1)	446 (96.3)	444 (91.9)**
Yes	56 (5.9)	17 (3.7)	39 (8.1)
ADL/IADL deficits, Mean ± SD	6.59 ± 5.45	5.52 ± 5.14	7.62 ± 5.54**
Number of chronic diseases, Mean ± SD	1.91 ± 1.36	1.69 ± 1.45	2.13 ± 1.23**

*ADL* Activity of daily living, *IADL* Instrumental activity of daily living, *MMSE* Mini-mental status examination

**P < 0.001, *P*-value by t-test or Chi-sqaure test for gender difference

^a^Income level is divided by tertiles, unit = 1000 KW

**Table 2:** Characteristics of receiving care and gender difference among dependent older adults in KLoSA (*N* = 946)

	Total N(%) or Mean (SD)	Men	Women	*P*-value for gender difference
		(Estimated % or LS mean)		
Primary main caregiver, %				
None (care deficit)	320 (33.8)	41.7	30.7	0.007**
Spouse	297 (31.4)	31.9	32.6	0.852
Children	140 (14.8)	10.3	19.0	0.004**
Other relatives, friends	91 (09.6)	8.7	8.7	1.000
Paid formal	98 (10.4)	7.4	9.0	0.469
Caregivers combinations (Main+additional), %				
None (care deficit)	320 (33.8)	41.7	30.7	0.007**
Spouse only	234 (24.7)	26.2	24.4	0.617
Spouse+unpaid informal	52 (05.5)	5.6	6.2	0.774
Spouse+paid formal	11 (01.2)	0.2	2.1	0.047*
Children only	84 (08.9)	6.3	11.9	0.032*
Children+unpaid informal	47 (05.0)	3.0	6.6	0.070
Children+paid formal	9 (01.0)	0.9	0.5	0.645
Other relatives, friends only	54 (05.7)	4.4	5.4	0.590
Other relatives, friends+unpaid informal	33 (03.5)	3.6	2.9	0.668
Other relatives, friends+paid formal	4 (00.4)	0.7	0.3	0.582
Paid formal caregiver only	54 (05.7)	5.5	3.5	0.290
Paid formal+unpaid informal	44 (04.7)	1.9	5.5	0.030*
Number of caregivers (*n* = 626), mean	1.40 (0.68)	1.317	1.485	0.029*
Caregiving days per month (*n* = 626), mean	24.51 (15.63)	25.59	21.25	0.006**
Caregiving hours per day (*n* = 626), mean	5.04 (4.97)	4.43	4.61	0.673
Payment to caregivers (*n* = 626), %	66 (7.1)	5.6	5.5	0.964
Cost for caregiving^a^ (*n* = 66), mean	48.48 (53.44)	35.13	38.57	0.887
Cost of own paid^a^ (*n* = 66), mean	24.80 (40.72)	24.34	14.13	0.446
Cost of family paid^a^ (*n* = 66), mean	39.01 (06.92)	10.80	24.43	0.517

*P < 0.01, **P < 0.001 ^a^ unit = 10,000KW; Estimated %, LS means and *P*-value were calculated by generalized linear model and ANCOVA for gender difference adjusting for age, marital status, living arrangement, region, household income, educational level, self-rated health, cognitive function, ADL/IADL deficits, number of chronic diseases, health insurance type, and long term care service usage

The second part of the data analysis addressed the second research objective, the relationship between marital and socioeconomic status and care received, again by gender. We performed the same analysis process with the gender differences above: we created a generalized linear model for binary outcomes (who is caregiver) and performed ANCOVAs for continuous numeric outcomes (how many hours, how many caregivers and how much payed, etc.) to examine the association of marital status, socioeconomic status (including equivalized household income tertile, personal income tertile, and educational level) and residential region with type and amount of formal and informal care received. Because educational level, family size and region had no significant association with any dependent variable, and because the results by personal income level were not different to those by household income, the results were presented only according to marital status and equivalized household income tertiles in Tables 3 and 4, respectively. To examine the gender difference of association, we tested the interaction effects of gender and marital status, and gender and socioeconomic status, on care received. Sensitivity analysis was conducted to check the reliability of the results. The level of functional limitation was divided into IADL disability, one to three ADL items disability, and four or more ADL disability groups. The significance of gender, marital status and household income of care deficit was similar in each functional status group, thus the reliability of the present analysis results was proved.

**Table 3:** Different receiving care by marital status among dependent older adults in KLoSA (*N* = 946)

	Marital status (Estimated % or LS mean)		*P*-value for marital status	Sig. of gender interaction
	Married	Widowed, divorced, separated, or others		
Primary main caregiver, %				
None (care deficit)	32.8	42.6	.018	**
Spouse	51.3	–		
Children	6.0	29.9	< 0.001	**
Other relatives, friends	2.8	19.2	< 0.001	
Paid formal	7.0	10.3	.147	
Caregivers combinations (Main+additional), %				
None (care deficit)	32.8	42.6	.018	**
Spouse only	39.1	–		–
Spouse+unpaid informal	9.8	–		–
Spouse+paid formal	2.4	–		–
Children only	4.4	17.5	< 0.001	
Children+unpaid informal	1.7	10.3	< 0.001	
Children+paid formal	0.0	2.1	.008	
Other relatives, friends only	2.0	10.1	< 0.001	
Other relatives, friends+unpaid informal	1.0	7.4	< 0.001	
Other relatives, friends+paid formal	0.1	1.8	.002	
Paid formal caregiver only	3.2	6.9	.044	
Paid formal+unpaid informal	3.8	3.4	.800	*
Number of caregivers (*n* = 626), mean	1.41	1.42	0.848	*
Caregiving days per month (*n* = 626), mean	23.83	22.10	0.282	*
Caregiving hours per day (*n* = 626), mean	4.87	4.00	0.054	
Payment to caregivers (*n* = 626), %	5.2	6.3	0.584	
Cost for caregiving^a^ (*n* = 66), mean	45.42	29.77	0.467	
Cost of own paid^a^ (*n* = 66), mean	23.80	11.54	0.305	
Cost of family paid^a^ (*n* = 66), mean	21.62	18.23	0.856	

*P < 0.01, **P < 0.001, ^a^ unit = 10,000KW; Estimated %, LS means and *P*-value were calculated by generalized linear model and ANCOVA for marital status difference adjusting for age, gender, living arrangement, region, household income, educational level, self-rated health, cognitive function, ADL/IADL deficits, number of chronic diseases, health insurance type, and long term care service usage

**Table 4:** Different receiving care by household income level among dependent older adults in KLoSA (*N* = 946)

	Household income (Estimated % or LS mean)			*P*-value for income difference	Sig. of gender interaction
	Low	Middle	High		
Primary main caregiver, %					
None (care deficit)	41.4^a^	38.7^a^	29.1^b^	0.015	*
Spouse	32.1	32.1	32.6	0.992	
Children	16.1	12.5	14.9	0.429	*
Other relatives, friends	5.1^a^	8.4^a^	12.5^b^	0.022	
Paid formal	5.3	8.3	10.9	0.077	
Caregivers combinations (Main+additional), %					
None (care deficit)	41.4^a^	38.7^a^	29.1^b^	0.015	*
Spouse only	27.5	25.8	22.7	0.469	
Spouse+unpaid informal	3.7	4.8	9.0	0.057	
Spouse+paid formal	0.9	1.6	0.9	0.652	
Children only	10.8	8.5	7.8	0.528	
Children+unpaid informal	4.5	2.8	6.8	0.112	*
Children+paid formal	0.8	1.2	0.3	0.548	*
Other relatives, friends only	2.7	4.8	7.2	0.116	
Other relatives, friends+unpaid informal	1.9	3.2	4.9	0.238	
Other relatives, friends+paid formal	0.6	0.5	0.5	0.986	
Paid formal caregiver only	1.5	5.1	7.0	0.022	*
Paid formal+unpaid informal	3.8	3.2	4.0	0.867	*
Number of caregivers (*n* = 626), mean	1.36	1.34	1.52	0.061	*
Caregiving days per month (*n* = 626), mean	22.00	22.02	25.35	0.083	*
Caregiving hours per day (*n* = 626), mean	4.24	4.26	5.06	0.160	
Payment to caregivers (*n* = 626), %	3.0	6.0	8.0	0.120	
Cost for caregiving^c^ (*n* = 66), mean	24.21	37.13	53.75	0.544	
Cost of own paid^c^ (*n* = 66), mean	5.42	17.44	32.34	0.201	*
Cost of family paid^c^ (*n* = 66), mean	18.80	19.70	21.41	0.994	

*P < 0.01, ^c^unit = 10,000KW; Estimated %, LS means and *P*-value were calculated by generalized linear model and ANCOVA for equivalized household income difference adjusting for age, gender, marital status, living arrangement, region, educational level, self-rated health, cognitive function, ADL/IADL deficits, number of chronic diseases, health insurance type, and long term care service usage; ^a,b^Results from contrast test

## Results

### Sample characteristics

The sample characteristics are shown in Table 1. The dependent older adults included in this study ranged in age from 54 to 108 years, with an average age of 78.0 years. All characteristics, except health insurance type, varied according to gender, with women being significantly older and having a lower level of education, lower household income, and poorer health. The men were more likely to be married (90.1%) than the women (35.0%). This huge gap of marital status might induce that women in this sample are significantly older than men.

### Care received

Table 2 describes the care received from formal and informal caregivers by gender. Regarding the care deficit, 33.8% of respondents who were dependent or disabled in at least one ADL/IADL domain had no caregiver. Regarding the current main caregiver, most respondents received it from their spouse, followed by from one of their children and then by paid formal caregivers. When the spouse was the primary caregiver, there were fewer additional caregivers. Spouses were most likely to be the sole caregiver, and 5% of dependent older adults were cared for by both their spouse and other family members. The estimated rate of respondents receiving care only from paid caregivers was 5.7%.

Elderly dependents received care from an average of 1.4 caregivers simultaneously, and for an average of 25.4 days per month and 5.0 h per day. The highest number of caregivers for a single respondent among our sample was five. The fees paid by family members to caregivers were greater than those paid by the cared-for person.

The greatest difference between men and women was seen in the care deficit which means they did not have any caregivers. Nearly 42% of disabled older men and 32% of women, after adjustment for all other covariates including health status, had no caregivers. In an additional analysis, we checked the care deficit for people who have ADL disability (one or more ADL items independent, *n* = 366), 21.2% of ADL disabled older men and 8% of women had not caregivers. The gender gap in care deficit is similar for less and more serious disability (data not shown).

There was no gender difference in the rate of respondents listing their spouse as the main caregiver, but women were more likely to receive care from children than men. The proportion of respondents receiving care from both their spouse and paid formal caregivers was greater in women than men. Having a paid formal caregiver as the main caregiver and receiving supplementary informal care from a family member was more frequently reported by women than men. Women also received care from a greater number of caregivers than men. The total number of hours of care received per day was similar between men and women, but men received care on 4.3 more days each month than women.

We next examined the association between marital status and care received (Table 3). A difference was detected in care deficit: 42.6% of the respondents without spouses did not have any caregivers, compared with only 32.8% of their married counterparts. Widowed, divorced, and separated older adults were more likely to receive primary care from their children, other relatives and friends than were their married counterparts. The likelihood of having more than one type of caregiver also varied by marital status, with the results being similar to those described above for primary caregivers. One interesting result was that married older people were less likely to receive care only from paid formal caregivers versus their single counterparts.

**Fig. 1:**
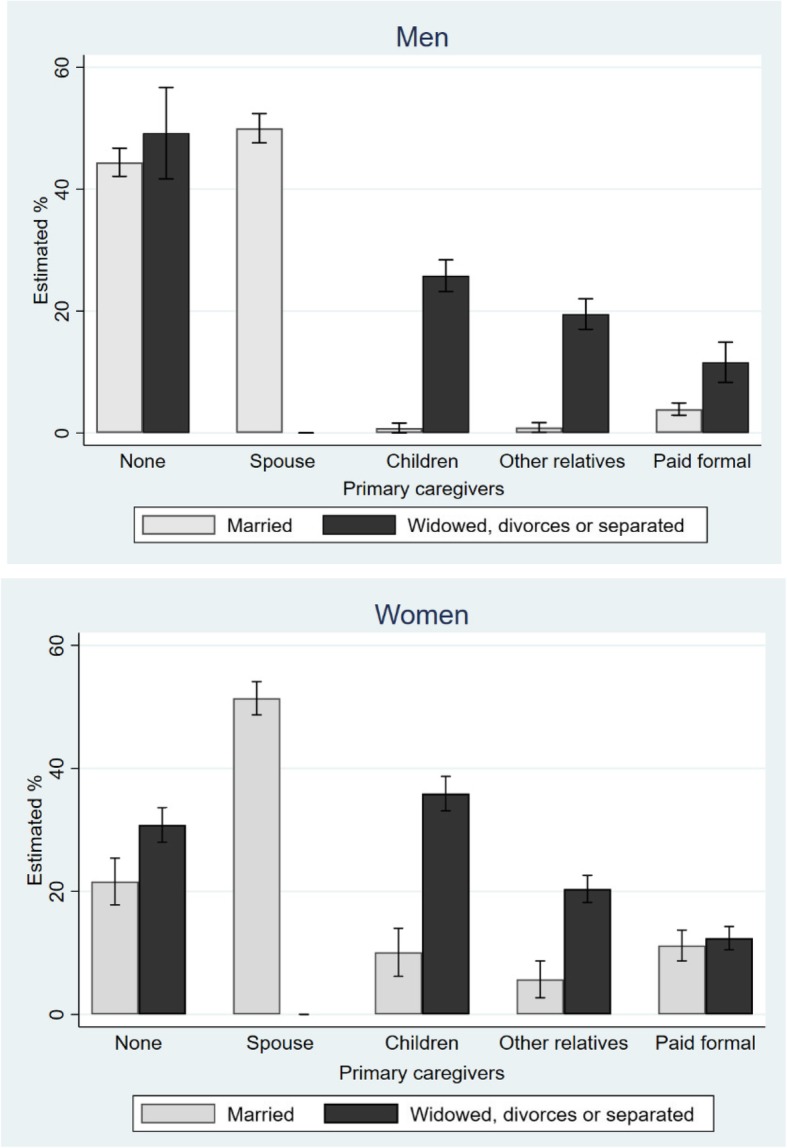
Estimated % of primary caregivers by marital status in Korean older men and women with disability

An interaction between marital status and gender was detected for the categories of care deficit, children as primary caregivers, type of paid or unpaid family caregivers, number of caregivers, and days of care per month. Because the care deficit and type of primary caregiver differed significantly by marital status for both genders, an estimated rate of primary caregiver according to marital status separated by gender was presented in Fig. 1. This confirmed the magnitude of the gender difference in the effect of marital status on the type of primary caregiver. Among the women, those who had no spouse had more care deficit and received more care from their children and other relatives and friends, while for the men, the estimated rate of care deficit was not associated with marital status even the rate was highest among men (49.2%). And the rate of paid formal care was significantly greater for unmarried versus married men.

The association between household income and care received among dependent older adults is presented in Table 4. A difference in type of primary caregiver by household income was also seen; the respondents in the highest household income tertile were more likely to receive care from any source than those in the lowest and middle tertiles. Respondents in the highest household income tertile reported receiving care from other relatives, friends, or neighbors more so than those who were in the middle or lower income tertile. No influence of income level on any other dependent variable was detected.

**Fig. 2:**
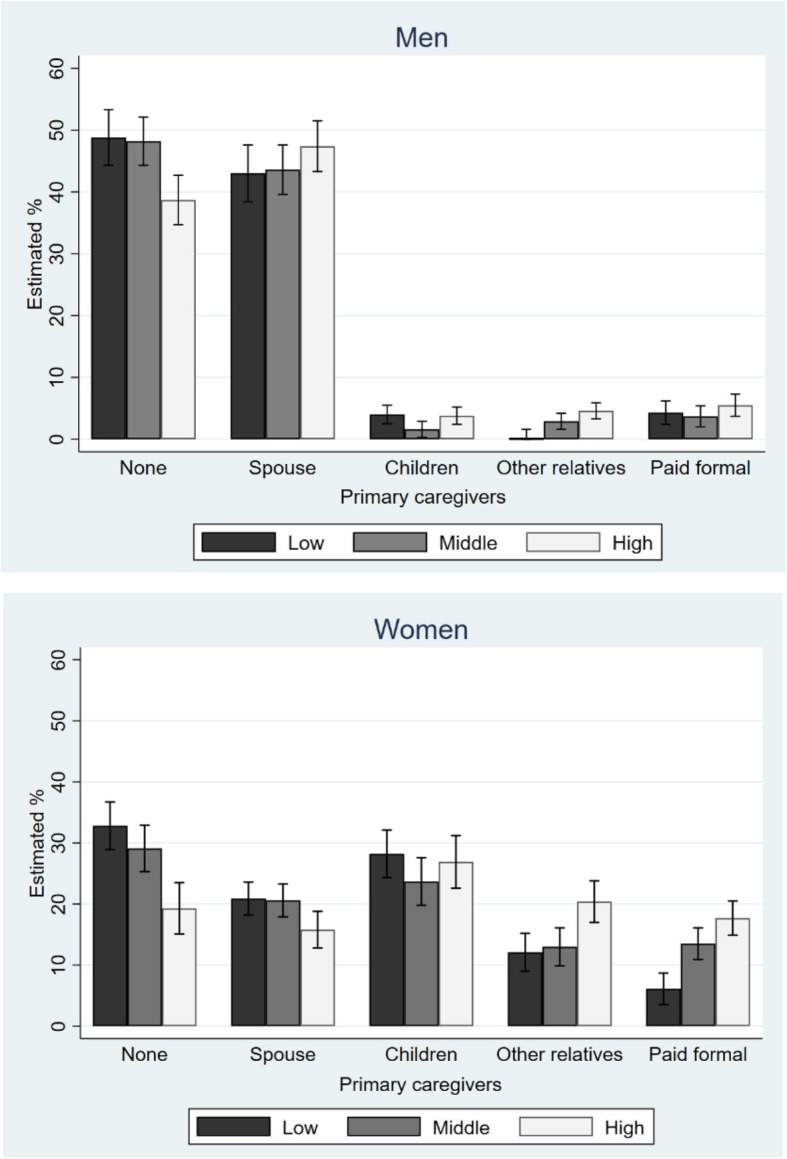
Estimated % of primary caregivers by equivalized household income in Korean older men and women with disability

The interaction effect of gender and income was significant for care deficit and the type of primary caregiver (*P* < 0.001). Figure 2 shows the adjusted results regarding the type of primary caregiver including care deficit, for both men and women, according to household income. The women in the highest income tertile received significantly more care from paid formal caregivers, whereas for men, the main caregiver was the spouse (with a similar pattern seen for all household income levels).

## Discussion

According to this study of care in South Korea, one-third of disabled older adults do not receive formal or informal care from any source. Both men and women primarily received care from their spouse; in contrast, children more frequently provided assistance to their mothers than their fathers. When a husband was a caregiver, he was more likely to have paid formal services. Men received 4.3 days more care than women per month. Marital status had a great influence on care type and resources. Care deficit was more frequent in men than women, with the rate being highest among older men without a spouse. In terms of socioeconomic status, dependent older people with high household incomes were more likely to receive formal or informal help than those in other income brackets. A difference in the rate of use of paid formal caregivers according to household income was detected in women.

Although studies of older persons living in their own homes suggest that men use informal care services more so than do women (e.g., Wan and Arlingm, 1983) [24] and that women were more likely than men to report unmet need for assistance with household tasks [13], we observed a higher rate of use of both formal and informal services by the women in our cohort. In a Malaysian study, men were also more vulnerable to unmet need [25]. This suggests the importance of examining gender differences in a variety of social contexts to determine the impact of such differences on the receipt of formal and informal care.

According to traditional Korean mores, even health care professionals are restricted in the amount of physical contact they should have with patients of the opposite sex. Older Korean men may be not easy to find caregiver either inside or outside of their family, because of their high dependence on their spouses. Interestingly, primary caregivers for married men could be clearly divided into two types (Fig. 1): those without caregivers or spouses as caregivers. Other types of caregivers were relatively rare compared to women. Older men are reluctant to use care services because they are afraid to receive care from strangers [26]. Individual gender role identity continues to change throughout life. Especially for men, they emphasize only “healthy” characteristics, so if males show signs of passiveness, enervated, and lack of self-confidence, they may be more negative than females. Male sex role negatively affects an individual’s developmental process adaptation as much as female gender roles [27, 28]. In the aspect of care-giving and care-receiving for the elderly, such toxic effects of feminity and masculinity are evident in both men and women.

Average days of care received per month was higher in men than women even under the similar total hours of care received by both men and women (i.e., the intensity of care was the same for both genders). One of possible interpretations of this gender difference concerns task specificity, where, women may be the primary recipients of assistance for specific IADLs, such as using telephone and the public transportation, counting money, and other tasks that do not require close and continuous contact between the caregiver and receiver [4]. Even though we adjusted the ‘care received’ data by health status and functional limitations, we did not consider gender differences in the types of tasks for which help was needed. Self-rated health, number of chronic diseases, ADL and IADL scores, and cognitive functions (as rated by the MMSE) can be assumed to index the need for care to the same extent. The optimal source of assistance for various ADLs and IADLs depends both on the nature of the task and the characteristics of the group or individual providing the help. In the light of task specificity, we can also interpreted that men may have more difficulty in tasks that must be completed every day (for example, preparing meals, cooking, and doing laundry). This study further showed that a combination of care from the spouse and a paid formal source was more frequently seen among the women. This shows that elderly women prefer to use formal care services even if they have a spouse [26, 29]. Male caregivers have difficulty in providing physical care (e.g., for toilet use and bathing) and assistance with housework (e.g., cooking). Physical care might also be resisted by the care recipient. Male caregivers have fewer opportunities to talk to other family members or friends about care-related issues than women, and tend to be more reluctant to share their burden. This is supported that the number of caregivers was higher among women. Studies of older persons living at home suggest that men make greater use of assistance from informal sources, whereas women tend to make greater use of assistance from formal providers, such as paid homemakers and home health aides. But this reflects the longer life expectancy enjoyed by women, such that older men are more likely to have spouses who are still alive, while women are more likely to become widowed and thus be reliant on external help. One previous study showed however, this difference in caregivers according to gender was observed among married couples [29]. Male caregivers are reluctant to perform “feminine jobs” (e.g., cooking, household chores, laundry, etc.) and so are more likely to seek formal help.

A gender difference was also seen in terms of the likelihood of the primary caregivers of the respondents being their children. Children more frequently provided assistance with ADLs/IADLs to their mothers than their fathers. This can be interpreted as showing that women are more dependent on care from their children when they are widowed. Older women are more likely than men to maintain relationships outside of their spouse, such as with their children and close friends [30]. In Korean society, older women not only feel more of an obligation to take care of their children, but must also look after their grandchildren, according to requests from their employed sons and/or daughters [31]. When the mother becomes frail and dependent, support exchanges dependent on a past or anticipated future exchange become more apparent and important [32]. This contradicts the study results of Nishi (2010) claimed that survival “penalty” for older Japanese women are cared for by their daughters-in-law [33]. In this study, Japanese male elders receiving care from daughters-in-law tended to live longer than those receiving care from their spouses. However, paradox gains that women more likely to be widowed so they receive care from daughter-in-law more, while men are fundamentally rare such cases that they receive care from their daughter-in-law. Based on our KLoSA dataset, daughter-in-law as a primary caregiver were only 4 cases (1.1% of total) for older dependent men.

Household income influences patterns of caregiving in this population. The financial resources of a family are closely related to their access to care. There may be few resources available to pay a caregiver, other family members may have disabilities, and an adult family member may need to work and be unable to take on a caregiving role. Indeed, in the highest income level in this study, the likelihood of the major caregivers being other relatives and friends was high, suggesting that the rate of recruitment of informal caregivers might vary by income. A lack of Information on formal help (from long-term care insurance services) may also have affected this trend. No difference was detected in the cost of care, days of care per month, or hours of care per day according to household income. The total amount paid cost to caregivers was not different by household income. This is because care is mostly provided by an informal caregiver, regardless of the cared-for person’s economic situation.

Among the women in our study, the major caregiver was more likely to be a paid formal caregiver when the household income was high. This is an important result, because it suggests that women in the lowest household income level tend not to be cared for primarily by a paid caregiver, although the situation is not the same for men. Older Korean women are highly dependent on their children in economic terms, and tend to adhere to family decisions rather than their own preferences [34]. Sometimes, such women avoid using formal care services to avoid placing any economic burden on their children [18]. According to our analysis, this tendency was more pronounced in the women in low-income households. In addition, the burden imposed by an older family member requiring care is greater in the context of the Confucian culture that older generations were exposed to, which views the necessity of entering a long-term care facility to be the result of the immorality of the children [30, 32]. Older women in Korean culture, fearing that shameful accusations of disobedience might be labeled at their child, might conceal their care requirements or forgo care to ease the burden on their children [26]. For example, the well-known Japanese novel ‘The Ballad of Narayama’ (1956) by Shichirō Fukazawa, and one of the most renowned Noh plays “*Ubasute-yama*” (literally meaning “Dump the Grandmother Mountain”), were based on observations of this phenomenon throughout East Asian countries. In ancient Korea and Japan, old women in some villages were expected to go into the mountains and starve themselves to death voluntarily to relieve their families of the pressures of caring for them; usually, their husbands were already dead.

Other independent variables that showed significant associations were age, functional status, and self-rated health. As age increases, the number of caregivers increased, and the probability that children were primary caregivers has also increased. Unlike the previous studies which have found association between the educational level and the type and quantity of care received, we could not find association with any dependent variable. The current older generation had fewer opportunities to receive a normal education in their youth, because they were in school or were young adults during the period of Japanese colonialism (1939–1945) and the Korean war (1950–1953). Only about 7% of Korean older adults graduated college or university in 2016 [35]. This homogenous low educational level might induce low association between care receiving outcomes and educational level. Korea launched long-term care insurance for the elderly in 2008, and increased access to home care services and long-term care facilities may have influenced the caregiving and care-receiving rates. However, long-term care insurance is available only for those with a severe disability, such that many care needs of elderly persons must be met by family members. Only 6% of our study population with disabilities had enrolled to national long-term care insurance. It is also notable that more investment was made in the development of facility-based versus home care services: in 2013, 48.2% of long-term care expenses were attributable to home-based care, and 51.8% to institutional care. For the 5-year period from 2009 to 2013, institutional care expenses increased by 8.5%, while home-based care service expenses decreased [36]. In the Korean long-term care insurance program, care allowance is available for family caregivers as salaried type, but the proportion of such claims among all long-term care claims is small (14%), and only half level of payment of formal caregiver is provided to family caregivers [37]. Even with universal long-term care insurance, serious inadequacies and inequities in care remain.

Family resources that have been in charge of informal care are gradually disappearing. Koreans work the longest hours of any Organization for Economic Cooperation and Development (OECD) country: the average Korean worker works 2069 h per year, which is 305 h per year longer than the average—1746 h per year—among the 35 OECD member countries [38]. Those who were still working in the 1990s witnessed many senior employees being laid off during the national economic crisis that occurred under the International Monetary Fund (IMF)-supported economic program, and as a result are concerned about their own job security [39]. The minimum retirement age is gradually decreasing in Korea, and many middle-aged men are seeking to re-enter the workforce or transition to self-employment [40]. Women are also taking up work in middle age to meet the rising costs of private education for their children. In this social context, care for the older adults showed inequality and this care inequality is manifested by various factors. The support policy for informal caregivers is much more needed in the long-term care system. More precise monitoring and more accessible long-term care services is needed so that there are no elderly people who do not receive and neglected the care they need. In order to increase the accessibility of paid formal care to low-income older women, it is necessary to reduce the burden of copayment.

Korea has entered an aged society with 14% of the total population, and GDP is the 12th highest among OECD members of the world in 2018. However, the public social expenditure compared to GDP is 10.4%, which is the lowest among the OECD countries (average 21% of OECD countries). The welfare expenditure of the elderly is only 2.2% of the GDP, which is insufficient compared with the rapid population aging. Ten years have passed since the long-term care insurance system was settled, but still socially and culturally dependent on family-centered care. In the socioeconomic and cultural contexts like Korea, examining the elderly ‘s deficit of care and inequalities in care could give similar messages to countries facing to both aging populations and increasing health disparity during economic development. This study has a policy implication that the long-term care system should be improved to meet the diversity of the older adults and to tighten the care services with customized care plans. It is necessary to strengthen daily support including a meal delivery, laundry or other house chores for older men. Care service should have strategy considering gendered pattern of service acceptance. Although not existing in the Korean long-term care system now, a care coordinating service by case management should be developed urgently. The cost burden of the older Korean women with low economic independence inside their family can be supported through enough information and appropriate counselling by care coordinator or case manager.

The limitations of this study and alternative explanations of the results should be considered. First, this study sample was based on community-dwelling older adults, which limits the generalizability of findings. There are important differences between community and institutional care settings in terms of the care needs and characteristics of individuals, as well as their rate of use of formal and informal care services. Even though the amount of caregiving cost was higher in married people, the difference was not statistically significant in this data. Married elderly might have more information and higher social resource utilizations; however, it needs further study on this relationship with larger sample dataset. In addition, because our analyses were cross-sectional, we are unable to infer any causality regarding the caregiving-related variables. Another limitation of this study was the source of information regarding the personal characteristics of the respondents. Many of the instruments used in this study were self-report measures. Reliance on self-reports, particularly regarding information on functional status, may result in inaccuracies and inconsistencies that limit the reliability of the results. Clinical evaluations of the presence and severity of mental illnesses and disability can be considered in the future research. And data from both caregivers and care recipients are needed to obtain more detailed information about formal and informal care in the context of caregiver–receiver dyads. There is also a need for research encompassing certain facets of unmet care needs that were not covered in the present study. For example, the perception of care and/or other psychological responses such as loneliness might be different from individual characteristics. Research is needed on the extent to which the needs for autonomy and control are met in the elderly, as well as on the factors that predict the likelihood of experiencing an unmet care need. In addition, we did not expand our analysis to older adults’ health and survival outcomes according to care-receiving inequality. Further investigation concerning the unmet care needs of individuals and its long-term health outcome is needed in various long-term care settings. As discussed above, our study did not consider the specific ADLs and IADLs that formal and informal carers helped with. Men and women show differences in dependence for different ADLs and IADLs. It is assumed that members of an individual’s informal social network will be most likely to assist with tasks that do not require close and continual contact, such as shopping and management of finances. However, the consistency of the results was confirmed by the sensitivity analysis comparing results by degrees of ADL and IADL disability.

## Conclusion

Unmet care needs were evident among the older men in this study, and this care deficit was greatest in those without a spouse; this may be interpreted as resulting from the gender segregation culture in Korea, which has a particularly large negative influence on elderly men. Household income also affects the likelihood of receiving care; especially, elderly women with a low household income are less likely to receive care primarily from a formal source. We identified inequalities in the care received among our cohort of older adults, and this will become an increasingly serious problem with the continued aging of Korean society. In societies where family care still accounts for a large proportion of all care, as in Korea, masculinity effect on care-receiving should be more considered in the further research as much as feminity of caregiving. Policies promoting a shared-care environment tailored to meet the needs, resources, and care preferences of individuals are needed.

## Data Availability

The datasets generated during and/or analyzed during the current study are available from the corresponding author on reasonable request.

## Abbreviations

ADL: Activities of daily living
ANCOVA: Analysis of Covariance
IADL: Instrumental Activities of Daily Living
KLoSA: Korean Longitudinal Study of Ageing
OECD: Organization for Economic Cooperation and Development
